# An NFATC4 phospho-switch links matrix stiffness to fibroblast fate

**DOI:** 10.1172/JCI209849

**Published:** 2026-09-01

**Authors:** Rebecca Shelley Frabotta, Purushothama Rao Tata

**Affiliations:** 1 Department of Cell Biology, Duke University School of Medicine, Durham, North Carolina, USA.; 2 Duke Regeneration Center, Duke University, Durham, North Carolina, USA.; 3 Division of Pulmonary, Allergy, and Critical Care Medicine, Department of Medicine, Duke University School of Medicine, Durham, North Carolina, USA.; 4 Center for Advanced Genomic Technologies, Duke University, Durham, North Carolina, USA.; 5 Duke Cancer Institute, Duke University School of Medicine, Durham, North Carolina, USA.

## Abstract

Fibrosis is driven by the activation of quiescent fibroblasts into contractile, matrix-secreting myofibroblasts, a transition governed jointly by biochemical signals and by the mechanical properties of the ECM. How the physical stiffness of tissue is converted into a durable transcriptional cell fate decision has remained poorly understood. In this issue of the *JCI*, Kadri et al. used global phosphoproteomic profiling of primary human lung fibroblasts across a defined stiffness gradient to identify phosphorylation of NFATC4 at residues S213/S217 as a mechanosensitive switch that is both necessary and sufficient for the fibroblast-to-myofibroblast transition. They validated these predictions in an independent transcriptomic dataset from patients with idiopathic pulmonary fibrosis, showing that NFATC4 expression increased with disease severity. Prior work has implicated NFATC4 activation in cardiac and hepatic fibrosis, suggesting that this single modification may serve as a convergence point for mechanical and cytokine signals across fibrotic diseases.

## The mechanical and biochemical microenvironment controls fibroblast state

The healthy alveolar ECM is soft (roughly 1–5 kPa), and the parenchymal fibroblasts it harbors remain quiescent at homeostasis (1). In idiopathic pulmonary fibrosis (IPF), progressive matrix deposition and collagen cross-linking stiffen the interstitium to 10–50 kPa and give rise to contractile, matrix-secreting CTHRC1^+^/ACTA2^+^ myofibroblasts that further amplify collagen deposition and propagate fibrotic remodeling (2, 3).

TGF-β is the canonical driver of this transition, but mechanical and biochemical cues are deeply intertwined. Stiff ECM amplifies TGF-β activation, and TGF-β in turn raises contractility and matrix secretion, stiffening the matrix further in a self-reinforcing loop (3–6). Crucially, stiffness does not merely permit differentiation, it can impose it: matrix stiffening activates RhoA/ROCK-dependent actin remodeling and MKL1 nuclear translocation to drive a fibrotic gene program (7), and sustained tension can hold cells in the myofibroblast state even without TGF-β, with cells relaxing back toward quiescence as local tension falls (8). Together, these findings suggest that fibroblast activation, once mechanically established, may persist independently of soluble cues such as TGF-β. How mechanical signals are ultimately translated into a committed transcriptional cell fate decision, however, has remained poorly defined.

## Charting the mechanosensing phosphoproteome

Protein phosphorylation is a central currency of mechanosensing: mechanical tension reshapes thousands of phosphorylation sites (hereafter referred to as phosphosites) at integrin-adhesion complexes, and these signals propagate inward to the nucleus to shape differentiation and gene expression (4, 9). Defining how specific phosphosites contribute to mechanosensing requires distinguishing those that are functionally decisive from the many that merely correlate with the response. This task demands both unbiased, proteome-scale discovery across a graded range of stiffness and site-specific functional validation.

**Figure 1:**
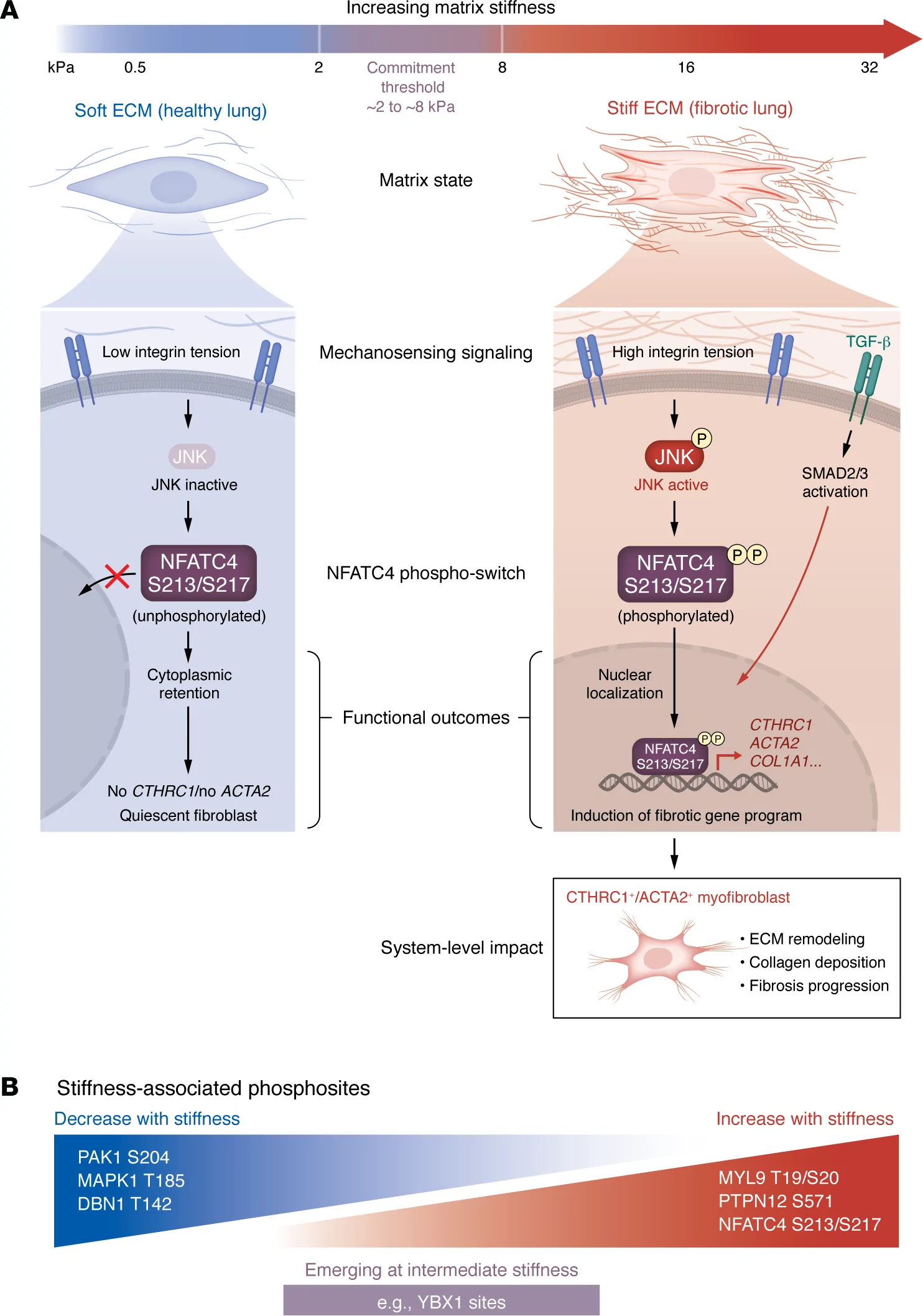
Matrix stiffness activates an NFATC4 phospho-switch gating the fibroblast-to-myofibroblast transition. Kadri et al. (10) used phosphoproteomic profiling in human lung fibroblasts cultured across a range of physiological matrix stiffnesses to identify mechanosensitive phosphosites involved in fibroblast differentiation. Among the candidates, NFATC4 emerged as a key regulator based on the strong association of its downstream targets with pathological fibrosis. (**A**) Increasing matrix stiffness promoted JNK-dependent phosphorylation of NFATC4 at S213/S217, enabling NFATC4 nuclear localization and induction of a fibrotic gene program. This stiffness-responsive switch drove CTHRC1^+^/ACTA2^+^ myofibroblast commitment, with TGF-β/SMAD2/3 signaling reinforcing the fibrotic state. (**B**) Kadri et al.’s study doubles as a resource for mechanosensitive phosphosites, adding substantially to those previously reported. This panel highlights select phosphosites spanning a range of roles, including cell adhesion, cytoskeletal reorganization, cell motility, and regulation of transcription.

In this issue, Kadri et al. applied mass spectrometry–based phosphoproteomics to map stiffness-dependent signaling in primary human lung fibroblasts plated on fibronectin-coated polydimethylsiloxane (PDMS) substrates that span a physiologically relevant range (0.5–32 kPa) (10) (Figure 1). By profiling five discrete stiffnesses rather than contrasting a single soft versus stiff condition, they captured signaling thresholds and regulatory transitions that a two-point design would miss. The resulting dataset is striking in scope: of 10,608 class I phosphosites, 1,631 were significantly regulated by stiffness, spanning essentially every major mechanotransduction compartment, including integrin-adhesion complexes, the actomyosin cytoskeleton, the nucleus, mitochondria, and the ER. A key threshold emerged at 2–8 kPa, above which cells committed to the CTHRC1^+^/ACTA2^+^ myofibroblast state; because this range overlaps the stiffness that is typically observed in early fibrotic tissue, even modest matrix remodeling may be enough to initiate activation. The dataset doubles as a standalone community resource, adding 656 previously unreported phosphosites and reporting time-resolved spreading experiments that resolve mechanosensing across time. Early attachment (10–30 minutes) was dominated by RNA processing and cytoskeletal remodeling, while later contractile phases (60–120 minutes) engaged Hippo-YAP1/TAZ, RhoA, and mesenchymal differentiation programs.

## NFATC4: a phosphorylation-gated checkpoint for myofibroblast fate

To move from this catalog to causal candidates, Kadri et al. applied a two-step filter: they first retained phosphosites with documented regulatory function in curated databases; then, within the transcription factor category, they kept only those phosphorylated at residues with defined roles in transcriptional control (10). Six high-confidence mechanosensitive transcription factors remained: EP300, TP53, NFATC4, FOXO3, YBX1, and RB1. NFATC4 stood out: its inferred regulon in IPF fibroblasts placed CTHRC1 and COL1A1 among its top induced targets, tying it directly to the CTHRC1^+^/ACTA2^+^ state that defines pathological fibrosis. NFATC4 is a calcium- and calcineurin-regulated transcription factor controlled by phosphorylation-dependent nucleocytoplasmic shuttling, with dephosphorylation promoting nuclear accumulation and activity (11). Its phosphorylation at S213/S217 rose progressively with substrate stiffness, and both residues were previously mapped as JNK substrates (12).

Using site-specific phosphomimetic (S213D/S217D) and phospho-dead (S213A/S217A) mutants, the authors showed that phosphorylation at these two residues is both necessary and sufficient to drive fibroblasts into the CTHRC1^+^/ACTA2^+^ state. The phosphomimetic mutant forced differentiation even on soft (0.5 kPa) substrates and without TGF-β — conditions under which control fibroblasts stay quiescent — whereas the phospho-dead mutant was unable to activate myofibroblast markers even on stiff matrix in the presence of TGF-β. Critically, overexpressing WT NFATC4 at comparable levels did nothing under any condition, establishing that fate is set by the phosphorylation status of NFATC4 rather than its abundance. This mirrors the well-established logic of the NFAT family, whose activity is gated by phosphorylation-dependent localization rather than expression level (11), and it identifies posttranslational control as the decisive layer linking mechanical input to cell identity.

Pharmacological JNK inhibition phenocopied the phospho-dead mutant in healthy and IPF patient–derived fibroblasts, consistent with JNK acting upstream of the NFATC4 phospho-switch and showing that the mechanism is conserved across healthy and already activated fibrotic cells. The particular state that NFATC4 controls matters as much as the switch itself. Across independent scRNA-seq studies, CTHRC1 has emerged as the defining marker of the pathological fibroblast population that expands in fibrotic lung, heart, and liver — a transcriptionally distinct, high collagen–producing subset largely absent from healthy tissue (2, 13). By sitting upstream of *CTHRC1* induction, the NFATC4 phospho-switch acts not as a regulator of generic activation but as a gatekeeper for entry into this specific, disease-driving cell state.

## Validation in human IPF

Importantly, these findings were extended to human disease. Using transcription factor regulon analysis of a published multicohort scRNA-seq atlas of pulmonary fibrosis (14), the authors found that NFATC4 activity is elevated in IPF fibroblasts compared with healthy controls (10). Among the six mechanosensitive transcription factors identified by phosphoproteomics, NFATC4 showed the strongest disease association, with its regulon encompassing 119 genes upregulated in IPF fibroblasts, including key mediators of fibroblast activation, ECM remodeling, and collagen cross-linking.

Immunofluorescence analysis of micro-CT staged IPF lung tissue showed NFATC4, CTHRC1, and ACTA2 increasingly colocalized as disease severity rose. This gradient is what one would expect if NFATC4-mediated mechanosensing is engaged progressively as tissue stiffens, and it anchors the S213/S217 axis as a disease-relevant regulatory node in the human lung. The conservation of phospho-dependent regulation in fibroblasts derived from patients with IPF is particularly notable. Despite their constitutively activated state, these cells exhibited the same sensitivity to both the phosphomimetic and phospho-dead mutants. This suggests that the JNK/NFATC4 axis remains operative after pathological reprogramming, and it is not merely an initiator of disease but a potentially tractable target in established fibrosis.

## Implications and open questions

The NFATC4 phospho-switch sharpens our understanding of how the physical microenvironment encodes cell identity in fibrosis. The finding that mimicking a single phosphorylation event substitutes for the stiffness cue driving differentiation on soft matrix, whereas preventing it blocks differentiation on a stiff matrix even in the presence of TGF-β, establishes this axis as a dominant, instructive regulator rather than merely a permissive one. Its relevance is unlikely to end at the lung. Work in cardiac and hepatic fibrosis already places NFATC4 downstream of calcineurin as a conserved profibrotic effector in mesenchymal cells (15, 16), and a recent spatial transcriptomic survey of interstitial lung diseases found NFATC4 activity enriched in CTHRC1^hi^ myofibroblasts (17). The JNK/NFATC4 S213/S217 switch may thus be a broadly shared mechanosensing node across fibrotic organs and a therapeutic target with cross-organ reach.

Several key questions emerge from these findings. Although supported by pharmacological evidence and prior studies, direct phosphorylation of S213/S217 by JNK in lung fibroblasts awaits formal validation by kinase assays. Likewise, how NFATC4 integrates with other mechanosensitive transcription factors that shape the pathological program, including RUNX1, remains to be determined (18). A central translational question is whether the JNK/NFATC4 axis can be therapeutically targeted in established fibrosis rather than only at disease onset. In addition, because this regulatory switch lies upstream of CTHRC1, it is important to determine whether NFATC4 directly occupies NFAT-binding elements at the *CTHRC1* locus or regulates its expression indirectly through downstream cytoskeletal programs. Distinguishing between these mechanisms will have important implications for therapeutic targeting of this pathway.

A complementary layer of this regulation lies in the noncoding genome. The transcription factors that read mechanical cues must ultimately act on *cis*-regulatory DNA, and those elements can themselves be mechanosensitive. Cosgrove et al. recently defined a class of stiffness-responsive enhancers, termed “mechanoenhancers,” whose chromatin accessibility and activity are tuned by ECM stiffness and that control fibrosis-associated genes including *CTGF* (also known as *CCN2*) and *MYH9* (19). Strikingly, epigenetic repression of the *CTGF* mechanoenhancer in primary lung fibroblasts blocked roughly 96% of the TGF-β–induced increase in CTGF and suppressed activation of the fibroblast-to-myofibroblast program, indicating that stiffness is encoded not only in the phosphorylation state of transcription factors but also in the chromatin state of the enhancers they occupy. These layers are likely interdependent: the S213/S217 phospho-switch governs whether active NFATC4 reaches the nucleus, while the accessibility of NFAT-bound enhancers near *CTHRC1*, *COL1A1*, and other myofibroblast genes would dictate whether nuclear NFATC4 can productively drive their transcription. Determining whether the phospho-switch and the mechanoenhancer landscape respond to the same stiffness thresholds and whether activated NFATC4 acts through these stiffness-gated regulatory elements is an attractive avenue for unifying signaling- and chromatin-level models of mechanically encoded cell fate.

A larger question is whether the switch behaves the same way in three-dimensional and in vivo settings, where cells read more complex and dynamic mechanical inputs. The PDMS substrates used in Kadri et al.’s study impose a fixed stiffness from the outset, whereas fibrosis in patients stiffens gradually over months to years and is spatially heterogeneous once established. It is therefore unknown whether NFATC4 phosphorylation reports a cell’s current mechanical state or instead encodes a stiffness history that becomes hard to erase once the CTHRC1^+^ program is locked in, a distinction central to whether fibrosis can be halted or reversed. Emerging materials could settle this: dynamically tunable hydrogels and piezoelectric or magneto-responsive substrates can change stiffness in real time beneath a living cell, unlike the static gels used here. Such platforms would let NFATC4 phosphorylation and nuclear translocation be watched directly as stiffness rises and would test reversibility by softening the substrate afterward to ask whether NFATC4 dephosphorylates, exits the nucleus, and releases cells from the CTHRC1^+^ state or whether the switch instead locks fibroblasts into a committed fibrotic identity. Pairing such substrates with stiffness maps of patient tissue, for instance, use of atomic force microscopy coregistered with NFATC4 phosphorylation status by immunofluorescence, could further test whether the 2–8 kPa threshold defined here matches the stiffness at the leading edge of fibrotic foci.

By identifying a discrete phosphorylation event that links matrix mechanics to pathological fibroblast fate, Kadri et al. recast a complex mechanobiological process long viewed through a biophysical lens as a defined signaling mechanism with clear therapeutic potential.

## Author contributions

RSF and PRT cowrote and edited the manuscript.

## Conflict of interest

PRT is an inventor on patents PCT/US20/53158 and PCT/US20/53164 and received research support from Calico Life Sciences, Ono Pharmaceuticals, and United Therapeutics.

## Funding support

This work is the result of NIH funding, in whole or in part, and is subject to the NIH Public Access Policy. Through acceptance of this federal funding, the NIH has been given a right to make the work publicly available in PubMed Central.

- NIH/National Heart, Lung, and Blood Institute research awards R01HL146557, R01HL160939, R01HL153375 to PRT.

## Version 1 09/01/2026

Electronic publication
